# Mixed Biopolymer Systems Based on Starch

**DOI:** 10.3390/molecules17010584

**Published:** 2012-01-09

**Authors:** M. Abd Elgadir, Md. Jahurul Haque Akanda, Sahena Ferdosh, Amid Mehrnoush, Alias A. Karim, Takahiro Noda, Md. Zaidul Islam Sarker

**Affiliations:** 1 Department of Life Sciences, Faculty of Pharmacy, Universiti Teknologi Mara, Shah Alam, Selangor, 42300 Malaysia; Email: saeedfood93@yahoo.com; 2 School of Industrial Technology, Universiti Sains Malaysia, Minden, 11800 Penang, Malaysia; Email: jahurulhaque@yahoo.com (M.J.H.A.); sfshila@yahoo.com (S.F.); akarim@usm.my (A.A.K.); 3 Department of Food Technology, Faculty of Food Science and Technology, Universiti Putra Malaysia, 43400 UPM Serdang, Selangor, Malaysia; Email: mehrnoush_amid@yahoo.com; 4 Hokkaido Agricultural Research Center, National Agriculture and Food Research Organization, Shinsei, Memuro, Kasai, Hokkaido 082-0081, Japan; 5 Department of Pharmaceutical Technology, Kulliyyah of Pharmacy, International Islamic University, Kuantan Campus, 25200 Kuantan, Pahang, Malaysia

**Keywords:** biopolymer systems, mixtures, protein, starch, rheological properties

## Abstract

A binary mixture of starch–starch or starch with other biopolymers such as protein and non-starch polysaccharides could provide a new approach in producing starch-based food products. In the context of food processing, a specific adjustment in the rheological properties plays an important role in regulating production processing and optimizing the applicability, stability, and sensory of the final food products. This review examines various biopolymer mixtures based on starch and the influence of their interaction on physicochemical and rheological properties of the starch-based foods. It is evident that the physicochemical and rheological characteristics of the biopolymers mixture are highly dependent on the type of starch and other biopolymers that make them up mixing ratios, mixing procedure and presence of other food ingredients in the mixture. Understanding these properties will lead to improve the formulation of starch–based foods and minimize the need to resort to chemically modified starch.

## 1. Introduction

It has long been recognized that starches derived from various botanical sources differ in the composition, structure and molecular order of their polysaccharide constituents (amylose and amylopectin), which could account for differences in their functionality. Starch owes much of its functionality to the fine structure and relative proportion of these polysaccharides, as well as to the physical organization of starch polymers into larger structural domains in the solid state [1].

Biopolymer mixtures are very important because of their great influences on food properties and formulation [2]. The various biopolymers that exist in aqueous mixtures of most food systems and their interaction in several ways produce different attributes which impact on quality, texture, and stability of the food systems. These structures play important roles in different quality attributes of food systems such as texture, flavour release and stability of many products, including dressings, desserts, jellies, and soups [3].

The blending of different types of flours and/or starches is an age-old technique used in the manufacture of various traditional food products. Simple binary mixtures of starch–starch or starch with other biopolymers (such as protein and hydrocolloids) are continuously being studied because such mixtures provide a new approach in producing tailor-made starch-based products. The possibility of formulating starch blends that behave like chemically modified starches; particularly with regard to pasting behavior was reported by Obani *et al.* [4]. Another objective is the partial substitution of more expensive or not so easily available materials by cheaper or more freely available indigenous flours or starches while maintaining or even improving product quality. In the context of food processing, the specific control and adjustment of the rheological properties is of significance in order to be able to regulate production processes and to optimize applicability, stability, and sensory properties of end products. Understanding such properties will lead to improvements in the formulation of starch-based foods.

Many researchers have reported that when two or more biopolymers are mixed together, the mixtures behaved differently from when they are present individually in a single phase [5,6,7,8]. This phenomenon is known as phase separation, which can be divided into two categories 1-associative phase separation whereby the first phase being enriched in both polymers, 2-segregative phase separation whereby each phase being enriched with one of the two biopolymers. In such mixed gel systems, interaction between polymers occurs. When the concentration of each polymer within the mixture of two or more polymers is higher than the critical gelation concentration, synergistic or antagonist effects can exist between the biopolymers. This phenomenon is generally due to excluded volume effects and water distribution between the phases [9].

The comprehensive review paper on mixed biopolymer system entitled “Starch–Biopolymer Interactions—A Review” was published in 1997 [10]. There have been many developments and papers published since then, not only on mixed starch system but also on starch–other biopolymers systems. Table 1 summarizes some studies conducted in the view of rheological characteristics of mixed biopolymer systems of starch base foods. The interest in studying properties of different biopolymer mixtures based on starch is reported within the past decade. This review, therefore, is intended to piece together the accumulated knowledge and review the trends and some pertinent findings on this subject. It will discuss the effect of mixing different polymers with starches and the effect of such process on the characteristics of the mixtures. The information would provide useful insight for food technologists/formulators to develop innovative starch-based products without the need to use chemically modified starch. Some suggestions for future research directions are also discussed.

## 2. Starch–Starch/Starch–Flour Systems

The utilization of starch mixtures in food products is not new, but can been traced to the dawn of history. Since ancient times, many types of traditional foods have been made from mixtures of different starches. For example, in the production of rice vermicelli, two different types of starches, usually corn and sago, are used together with rice flour to reduce the raw materials costs. Likewise, fish crackers are usually made from a mixture of tapioca and sago starches. The rationale underlying the use of starch mixtures most likely is due to desirable textural and sensory attributes conferred by the specific mixtures that cannot be obtained from any of the individual starches alone.

Several studies have been conducted based on different properties of starch-starch mixtures (Table 1). Different starch blends proposed as a simple substitute for chemically modified starches were reported by Ortega-Ojeda *et al.* [18], due to the increasing demand for natural food [4], for example, observed that blends of starches from multiple sources can possess some of the characteristics of chemically modified starches. Common corn, waxy corn, 50% high-amylose corn, wheat, potato, tapioca and rice are examples of such natural starches. It was reported that the characteristics of chemically modified starches could be due to interactions between solubilized portions of one starch with granules of other starches [4]. The study revealed that, with the exception of a wheat/tapioca combination, most of biopolymers blends exhibited very little retrogradation.

The functional properties of mixed starch system are governed by both the extent of gelatinization and retrogradation. It has been shown that the rate of retrogradation of starch gels on aging generally increases with increasing amylose content of the starch [19,20]. For starch mixture, the situation is potentially more complicated than for a single type of starch, since the morphology of starch granules themselves are not homogeneous, and the amylose/amylopectin molecules from each starch possess a definite ultra structure.

**Figure 1:**
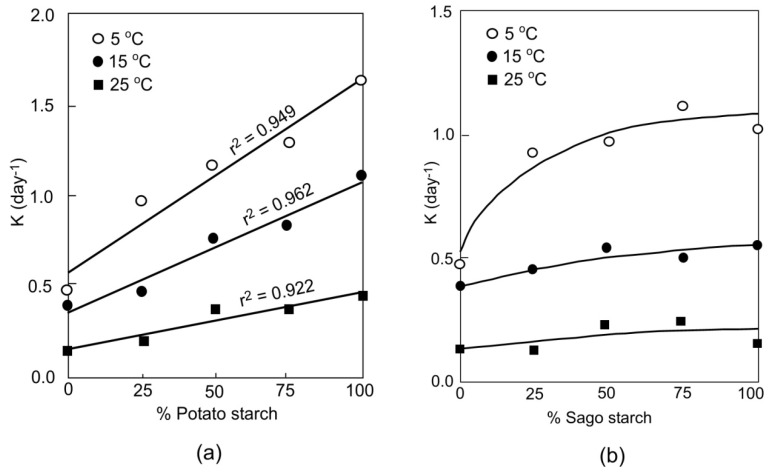
Rates of retrogradation (K) of (**a**) rice/potato starch and (**b**) rice/sago starch (1:2 dry starch/water ratio) gels at 5, 15, and 25 °C [21].

Using pulsed low-resolution NMR to monitor retrogradation of mixed rice starch with other starches (corn, potato, sago, and mungbean) in 1: 2 dry starch/water systems, it was found that in any particular mixture, the rate of retrogradation fell between those of the individual component starches [21]. In the absence of any significant interaction between starches, the rate of retrogradation would be expected to be linearly related to the level of substitution of one starch with another starch. This appears to be true for a mixture of mungbean + rice starches [21], potato + rice starches [22] (Figure 1a), and rice + waxy corn maize [22]. Similar additive effect of starch mixtures such as mixtures of potato and waxy maize as well as potato and barley was reported [23].

**Table 1:** Selected studies on mixed starch-biopolymer systems.

Type of starch mixture	Experiment	Observation	Ref.
β-glucans–starch mixtures	Four β-glucan preparations, *i.e.*, curdlan (CL), oat (OG), barley (BG) and yeast (YG) β-glucans, were compared for their effects on the gelatinization and retrogradation of rice starch (RS).	● The addition of any of these β-glucans significantly increased the peak, breakdown, final, and setback viscosities of RS, whereas the pasting temperatures were significantly decreased by OG or CL addition, but were unaffected by BG or YG addition. ● β-glucans had a negligible effect on the onset (*T*_o_), peak (*T*_p_), and conclusion (*T*_c_) temperatures but slightly decreased the gelatinization enthalpy ( 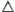 *H*_1_) of RS. Storage of all the gels at 4 °C resulted in a marked decrease in the *T*_o_, *T*_p_, *T*_c_, and melting enthalpy ( 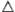 *H*_2_) values.	[11]
Milk protein–polysaccharide mixtures	Concentration of whey protein concentrate (WPC) at 1.0% and the pH 7.0 were mixed with commercial polysaccharides (PS) in concentration of 0.0–1.0%. Interactions between WPC and PS in the aqueous phase were evaluated.	● The results revealed differences in the molecular dynamics of mixed systems. ● The nature of the interactions between WPC and PS depended on the PS type, its relative concentration in the aqueous phase and also on the two WPC fractions. ● Whey protein concentrate/sodium alginate (WPC/SA) mixed systems were distinguished by a tendency to protein aggregation in the aqueous phase and their segregation into separated microdomains. ● WPC/λ–carrageenan (WPC/λ–C) mixed systems showed high degree of attractive interactions over the whole range of concentrations.	[12]
Hydrocolloid–flour mixtures in batter systems	The functionalities of hydrocolloid–flour mixtures in terms of the thermal properties of their resulting batter systems were investigated. Gelatinization temperature (*T*_G_), total enthalpies of gelatinization (Δ*H*_G_), glass transition temperature (*T*_g_), melting peak temperature (*T*_m_), and total melting enthalpies (Δ*H*_m_) and the effects of different thermal processes such as cooking-freezing-thawing (CFT) and freezing-cooking (FC) on thermal properties of the various batter systems were determined.	● The different thermal processes did not significantly affect either *T*_G_ or Δ*H*_G_ of batter systems, but they influenced the glass transition behavior and the Δ*H*_m_ of batter systems. ● The thermal processes also showed different effects on the batter systems containing different hydrocolloids such as methylcellulose (MC), carboxymethyl–cellulose (CMC), and xanthan gum (XG). ● The hydrocolloids shifted *T*_G_ upwards, depressed *T*_g_, and increased *T*_m_ of batters. ● The effect of these hydrocolloids on glass transition temperature was more pronounced in raw samples (FC process) than in cooked samples and increased with increasing levels of CMC and MC used in the formulations. ● Batters with MC showed increased Δ*H*_m_ for all the thermal processes.	[13]
Non-waxy rice starch–hydrocolloid mixtures	The swelling and pasting properties of non-waxy rice starch–hydrocolloid mixtures were investigated using commercial and laboratory-generated hydrocolloids at low concentration of (0–0.1%).	●Hydrocolloids enhanced the trough and final viscosity of rice starch dispersions. ● They also lowered peak viscosity of rice starch dispersions. ● Total setback viscosity appeared to be not affected by hydrocolloids at low concentration (0.05%). ● The hot and cold paste of the starch–gellan gum mixture exhibited the highest viscosity values.	[14]
Rice starch–β-glucan mixtures	Rice starch (*RS*)-β-glucan (BG) mixture were investigated as functions of mixing ratio and of storage time at concentrations of RS/BG = 6.0/0.0, 5.7/0.3, 5.4/0.6, and 5.0/1.0.	●An increase in onset (*T*_o_), peak (*T*_p_), and conclusion (*T*_c_) temperatures and a decrease in gelatinization enthalpy (Δ*H*_1_) with increasing BG concentration. ● Storage of the mixed gels at 4 °C resulted in a decrease in *T*_o_, *T*_p_, *T*_c_, and melting enthalpy (Δ*H*_2_). ● The retrogradation ratio (Δ*H*_2_/Δ*H*_1_) and the phase transition temperature range (*T*_c_−*T*_o_) of the mixed gels increased with storage time. ● BG addition also slowed the syneresis of the mixed gels. ● The added BG also retarded the development of gel hardness during refrigerated storage of the RS/BG mixed gels.	[15]
Rice starch–hydrocolloid mixtures	Dynamic viscoelastic and steady flow properties of the freshly prepared pastes of starch alone and starch–hydrocolloid mixtures; cellulose derivatives and carrageenans were determined after holding at room temperature (~25 °C) for 1 h.	● Increases in apparent pasting temperatures and peak and final viscosities in the following decreasing order were observed: Methylcellulose > carboxymethyl–cellulose for cellulose derivatives and λ– > í– > κ–carregeenan for carrageenans. ● Slight decreases in peak and final viscosities were observed when hydroxypropyl–methylcellulose was the hydrocolloid.	[16]
Corm starch–guar gum mixtures	Gelatinization behavior of corn starch was studied in the presence or absence of various guar gum samples with different molecular weights in order to clarify the difference in functions of each guar to starch.	● Guars with M_w_ values higher than 12.2 × 10^5^ g/mol shifted the onset of viscosity increase for the system to lower temperatures and increased its peak viscosity upon heating at a relatively low starch concentration. ● The earlier onset of viscosity increase was independent of M_w_ of guar, while the increase in peak viscosity was dependent on its M_w_. ● These guars shifted the onset of viscosity increase for the system upward, on the contrary, at a relatively high starch concentration (e.g., 15%).	[17]

A synergistic interaction was noticed when rice and sago starches are mixed [21] (Figure 1b). Similar synergistic effects for a mixture of rice and tapioca starches (1:2 and 2:1) and rice–hydroxypropylated potato starches were also reported by Yao *et al.* [22]. In both mentioned cases, higher retrogradation tendencies than the respective starch components in the starch mixtures were displayed. It was also observed that low temperature storage (5 °C) not only accelerates starch retrogradation, but also accentuates whatever interactions between the different starch polymers that lead to higher than expected retrogradation rates [21].

To explain the sysnergistic interactive effect of starch mixtures, an ideal gelatinized starch mixture system was defined as a fresh mixture of two separately gelatinized starch systems with the same moisture content [22]. This definition is called distribution of regional moisture content (RMC). It is assumed that no moisture redistribution occurs among swollen granules during gelatinization and retrogradation. The distribution of RMC for ideal mixture gel could be described as the sum of the proportional contributions of the two starch components in the mixture [22]. In actual mixture gels, which take place when two different types of starches are mixed at the granular level [22] during gelatinization process, the swollen granules from one starch may contain higher moisture content than the granules from another starch. However, the researchers found that the distribution of RMC in the mixture and the pulsed NMR signal (denoted by S’ in their study) of each individual granules in the system may also be different from those of the ideal gel mixture. Under this circumstance, it was concluded that the S’_mix_ value will not be calculated as the average value of S’_1_ and S’_2_ due to the proportion of two starch components, which might result in the non–additive nature of S’. This idea merits consideration and further investigation. Some researchers reported that at low starch concentration (20%), each individual component in the mixture independently gelatinized, whereas at higher starch concentration (50%) they did not [23].

**Figure 2:**
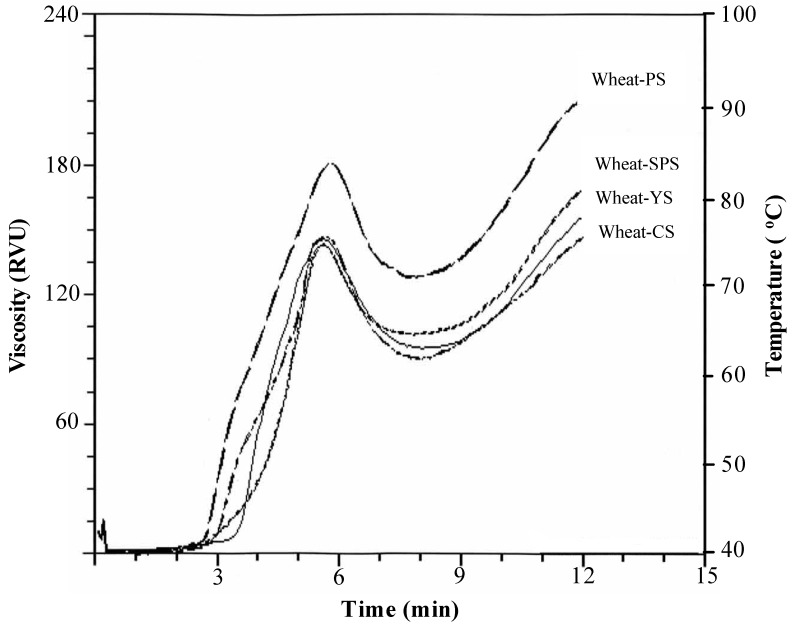
Typical RVA pasting curves for the mixtures of wheat–PS, wheat–SPS, wheat–YS, and wheat–CS at 30% starch in the mixture [24].

The pasting properties of mixtures of wheat flour mixed with tuber starches namely sweet potato starch (SPS), potato starch (PS), yam starch (YS), and cassava starch (CS), at 10 to 50% starch in the mixtures was reported by Zaidul *et al.* [24] (Figure 2). RVA was used in the measurement. A significantly (*P* < 0.05) increase in the peak viscosities was notice with increasing starch content in the mixtures. On the other hand, the control wheat showed too low peak of viscosity which also was out of ranges. Thus, it was found that combining starches with wheat flour for optimizing the viscosity was important processing practice. Similar finding of peak viscosity characteristics was reported in the blend of sago starch and wheat flour from 10 to 50% sago in the mixtures [25]. These trends were supported by similar study results for the mixtures of wheat flour and potato starches with varying contents of amylose and phosphorus [26]. The authors also observed at 40 and 50% PS, dramatic increase in the peak viscosity was noticed which attributed to the lower setback viscosities with 40 and 50% PS in the mixtures. The same phenomenon in the mixtures of sago–wheat and wheat–potato mixtures was observed by Zaidul *et al.* [25,26].

## 3. Starch–Protein Systems

Starch from cereals, roots and tubers and protein from meat, fish and other sources play major roles in food systems. When mixed in the system, they provide the desirable rheological and textural attributes of food. Starch contributes in physical properties of many food products such as confectionery, sauce, pudding, comminuted meat and fish products and several low–fat products [27]. The stability of food dispersions depend on protein–starch interactions in bulk solutions and at interfaces.

Mixing ratios of 0.15:1, 0.2:1, and 0.3:1 whey protein isolate (WPI)–wheat starch (dry weight basis) and combination of WPI and egg white protein were used by Yang *et al.* to study the rheological properties of starch and dairy ingredient-based food systems [28]. They found that the changes of storage modulus (*G*^′^) of starch–WPI mixtures during the cooling were different from that of starch gel alone. At a low WPI content (1:0.15), *G*^′^ decreased during cooling. When they increased the content of WPI to 1:0.2, *G*^′^ still decreased, but the reduction was not as much as that at the 1:0.15 levels. When more WPI (1:0.3) was added, *G*^′^ was more than that of pure starch gel at 85 °C. When temperature decreased, *G*^′^ initially decreased and at 65 °C, *G*^′^ started to increase. It was found that unlike hydrogen bonds, hydrophobic interactions are enhanced in warm temperature (40–60 °C) [29]. Therefore, when more WPI was added to the mixture, the effect of hydrophobic bonds on the gel during cooling from 65 to 30 °C, increased. The authors found that when egg white protein was used to replace 50% of the WPI, a decrease in *G*^′^ and increase in tan δ could be observed. However, there was a continued increase of gel strength due to hydrophobic interactions at warm temperature and hydrogen bond interactions at cold temperature, during the cooling. They concluded that WPI replacement for egg white protein might be beneficial in increasing elastic modulus and the rigid structure of bakery products during the later stage of baking and during cooling.

Bertolini *et al.* [30] reported that the addition of sodium caseinate (NaCN) to starch led to an increase in the peak viscosity for all starches with the exception of potato starch, which showed a reduction in peak viscosity (Table 2). The study also showed that NaCN increased final viscosity for all starches. In addition, NaCN did not alter peak temperature for wheat, corn and rice starches, while the presence of NaCN enhanced the peak temperature by 23 °C for potato starch. From differential scanning calorimetry data, the addition of NaCN resulted in definite increases in onset and peak temperatures for gelatinization.

A recent study was carried out to investigate the effects of starch/protein mass ratio (0.2–1.0) and different cooling rate (2, 4 and 6 °C/min) on rheological behavior of the composites [31]. The addition of starch significantly increased the *G*′ of flaxseed protein concentrate, which is similar to that of the addition of potato starch to acid milk [32] and addition of corn starch to soy protein concentrate [33]. Starch granule–soy protein and wheat protein interactions were investigated by Ryan *et al.* [34]. The study showed that the adsorption of proteins on starch at 0.2, 3.5 and 5.0 mg/mL protein solution in varying pH. Similar findings were reported in another study [35].

Various starches have been included in meat products such as surimi and low fat sausage for reducing cost and improving texture. Starch added in meat emulsions favors the formation of a more compact and stronger heat induced protein network [36]. Moreover, the addition of pre–gelatinized starch decreased the firmness and water holding capacity of surimi product as compared to the addition of native potato starch [37]. Starch is also used to improve the texture of low fat bologna [38], low fat frankfurters [39], and comminuted scalded sausages [40]. 

The effect of adding different starches (corn starch, high amylose and waxy corn starch, tapioca starch, potato starch, pea starch, mungbean starch, rice starch and sweet potato starch) to starch–meat complexes was investigated by Li *et al.* [41]. The authors found that as temperature was raised from 40 to 50 °C, *G*′ decreased slightly due to the temperature effect. At temperatures lower than 55 °C, the starch–meat complex exhibited a similar *G*′ to pork ham batter. The initial protein denaturation resulted in an increase in *G*′. Associated with the gelatinization of starch, there existed a dramatic increase in *G*′ above 60 °C.

**Table 2:** Average of RVA parameters for starches with and without sodium caseinate and their significance levels for the starch, caseinate and their interactions [30].

Starch/treatment	peak viscosity (RVU) ^a^			final viscosity (RVU) ^a^			Peak temp. (°C)	
		ΔPV ^b^			ΔFV ^b^			ΔPT ^b^
waxy corn	244			115			80	
waxy corn caseinate	393	149		208	93		83	3
potato	760			261			72	
potato caseinate	389	−371		362	101		95	23
cassava	281			168			88	
cassava caseinate	403	122		287	119		91	3
wheat	122			131			95	
wheat caseinate	148	26		269	138		95	0
corn	141			140			95	
corn caseinate	206	65		328	188		95	0
rice	96			159			95	
rice caseinate	123	27		251	92		95	0
	*P* levels ^c^							
starch	<0.001			<0.001			<0.001	
caseinate	0.0308			<0.001			<0.001	
starch × caseinate	<0.001			<0.001			<0.001	

^a^ Rapid Visco Analyzer units; ^b^ ΔPV, ΔFV, and ΔPT indicate differences between sodium caseinate-starch and starch systems for peak viscosity, final viscosity, and peak temperature, respectively; ^c^ Significance levels (*P* > *F* values) in RVA means for different starches, samples with and without sodium caseinate and their interactions.

The thermal processing of fish protein–starch systems was studied by Wu *et al.* [42]. The researchers observed significant rheological changes during heating. These changes could be due to sol–gel transformation of fish proteins and gelatinization of starches. It was observed that the addition of starch did not affect thermal transitions of fish proteins, as monitored by differential scanning calorimetry [43].

## 4. Starch–Hydrocolloid Systems

Polysaccharides or hydrocolloids have been studied by many researchers (Table 1). It was found that starch granule swelling or melting during gelatinization was highly influenced by the presence of hydrocolloids and synergistic interactions between hydrocolloids and starch [44]. In general, the viscosity of the mixed system was much greater than that of the starch alone, since most biopolymers are strongly hydrophilic and compete with the starch for water [17].

Starch–gum mixtures (6% w/w starch and 0.35% w/w gum) were studied using RVA in order to investigate the effects of guar and xanthan gums on pasting and rheological properties of native and anionic tapioca starches [45]. The result revealed that compared to the native tapioca starch (as control), additions of guar and xanthan gums resulted in significant (*P* ≤ 0.05) increases in peak, breakdown, setback and final viscosities, pasting temperatures and peak times (time to reach the peak viscosity), except for guar addition, in which the peak time was unaffected and xanthan addition, in which case setback viscosity decreased significantly (*P* ≤ 0.05). A similar tendency was also found for the anionic tapioca starch–guar gum mixture as compared with its control (anionic starch alone) [45]. 

The rheological description of galactoxyloglucan hydrocolloid (HXG) which contained galactose, xylose, and glucose in a 1:3:4 molar ratio was studied by Freitas *et al.* [46]. HXG was mixed with a high amylose (66%) and waxy corn starch. They found that the value of apparent viscosity at 1.5 s^−1^ was greater for both mixtures, the increase being the greatest after 20 h storage at 5 °C of high amylose and waxy corn starch solutions at a concentration of 25 g·L^−1^.

Rheological properties of rice starch–xanthan gum mixtures (5% w/w) at different xanthan gum concentrations (0, 0.2, 0.4, 0.6, and 0.8% w/w) were evaluated in steady and dynamic shear [47]. All the tested samples had highly shear-thinning behavior with values of flow behavior indexes (*n*) as low as 0.14–0.24. The *n* values decreased with increase in xanthan gum concentration. From these results, the authors found that the rheological behaviors of rice starch–xanthan gum mixtures were apparently dependent on the concentration of xanthan gum [47].

Increasing xanthan gum concentration from 0.2% to 0.8% caused an increase in the magnitudes of consistency index. This may be attributed to an increase in the viscoelasticity of the continuous phase in starch–gum composite systems due to the thickening properties of xanthan gum [48]. From these observations, it was found that the dynamic rheological properties of rice starch–xanthan gum mixtures were affected by the addition of gum and depended on the gum concentration [47].

## 5. Directions for Future Study

The increasing number of published papers on this subject signifies the potential of fabricating starch-based products by exploiting the complex interactions of starch–starch and starch–other biopolymers that give rise to diverse textural attributes. The rationale underlying the use of mixed biopolymer systems based on starch most likely is due to desirable textural and sensory attributes conferred by the specific mixtures that cannot be obtained from any of the individual starches alone. In addition, the future may see an emphasis on new methods of using starch rather than the proliferation of chemical derivatives. This means that research should be focused on physical processes or investigating possible synergistic/antagonistic relationships that alter starch characteristics, rather than using chemical modification.

Many of the studies involving mixed starch–based systems are empirical in nature and did not attempt to elucidate the underlying mechanism involved to explain the manifested changes in some functional properties of the system. Present knowledge appears inadequate to formulate a scientific basis for predicting the effects on retrogradation resulting from the blending of different types of native or modified starch. Systematic study using intensive numerical simulations (such as neural network) could be useful in order to build up various models of mixed polymer–starch system and assist the technologists to predict the behavior of such systems.

The synergistic interaction displayed by some starches in mixed systems merits further scrutiny as to the nature of interaction at the molecular level. In cases where the mixture exhibits an additive effect, it is presumed that the starch molecules (amylopectin) from both starches behave independently in the mixture. The rate of retrogradation of the mixtures can, therefore, be predicted by a linear relationship. For synergistic interaction, however, there could be some kind of physical (or possibly chemical) factors that play a role in accentuating retrogradation rate of the mixture, over and above the predicted additive value. The questions arising under this situation is: Just how exactly the granules from two different populations, containing amylose and amylopectin (from both starches) mix and interact within the composite system? There are apparently many factors (such as amylose/amylopectin ratio, chain length, degree of branching of the starch mixtures, degree of gelatinization, extent of starch granule swelling or disruption, phase separation) that can influence the course of such interactions.

Due to the diverse and complex nature of starch and other biopolymers, a broad range and variation in functional properties of the mixed biopolymers would be expected. From the data obtained so far, it is obvious that interactions (if any) between different starches and between starch and other biopolymers when blended are still not well understood. This is particularly so where the phenomenon of starch retrogradation is concerned. While the retrogradation tendencies of individual starches have been studied extensively, those of mixtures of starches have received little attention. Manipulation of the rate or extent of starch retrogradation by the simple expedience of blending different flours or starches is of technological importance to the food industry.

## 6. Conclusions and Future Outlook

The proper combination of starch–based mixed biopolymer systems provides a convenient approach in achieving desirable functional properties such as pasting, viscosity, viscoelasticity, thermal and textural functionality in the preparation of innovative food products. However, the actual mechanisms of interaction between the polymer constituents are unknown yet and warrant further investigation. An understanding of interactions of various components, especially amylopectin, may be critical to understand the retrogradation behavior of starch mixtures, hence manipulation of the property in formulating starch–based food products. Nevertheless, appropriate blending of different native starches may provide a simple practical avenue for manipulating, at least to some extent, the functional properties and final quality of a starch-based end-product. It is not impossible that starch–based mixture would find wider usage in view of increasing consumer demand for more natural product.
